# Supplementary material for: Exsolution trends and co-segregation aspects of self-grown catalyst nanoparticles in perovskites
Source: Nat Commun. 2017 Jun 28;8:15967. doi: 10.1038/ncomms15967 (PMC5493762; doi:10.1038/ncomms15967)
Supplement: Supplementary Information [file ncomms15967-s1.pdf]

Type of file: PDF
Size of file: 0 KB
Title of file for HTML: Supplementary Information
Description: Supplementary Figures, Supplementary Tables and Supplementary References

Type of file: PDF
Size of file: 0 KB
Title of file for HTML: Peer Review File
Description:

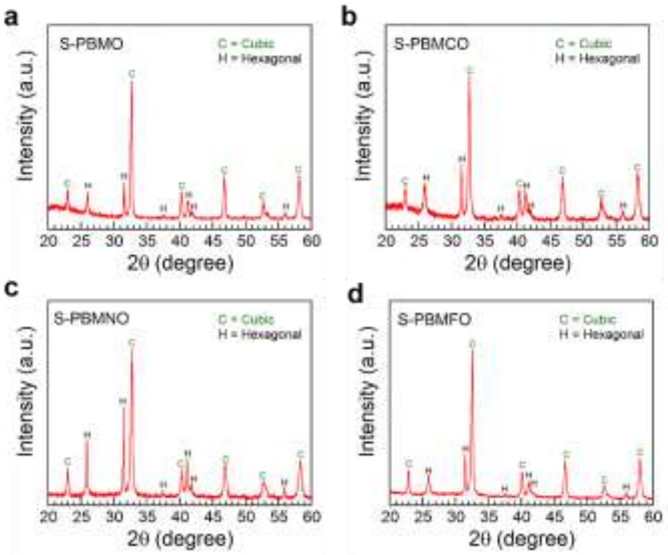

Supplementary Figure 1. X-ray diffraction patterns of (a) S-PBMO, (b) S-PBMCO, (c) S-PBMNO, and (d) S-PBMFO after sintering at 950 ^{o}C for 4h in air.

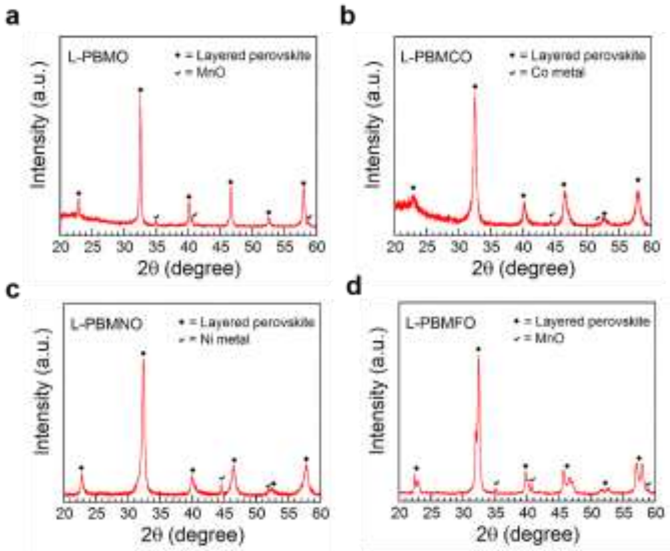

Supplementary Figure 2. X-ray diffraction patterns of (a) L-PBMO, (b) L-PBMCO, (c) L-PBMNO, and (d) L-PBMFO after reducing at 800 ^oC for 4h in humidified (3% H_2O) H_2.

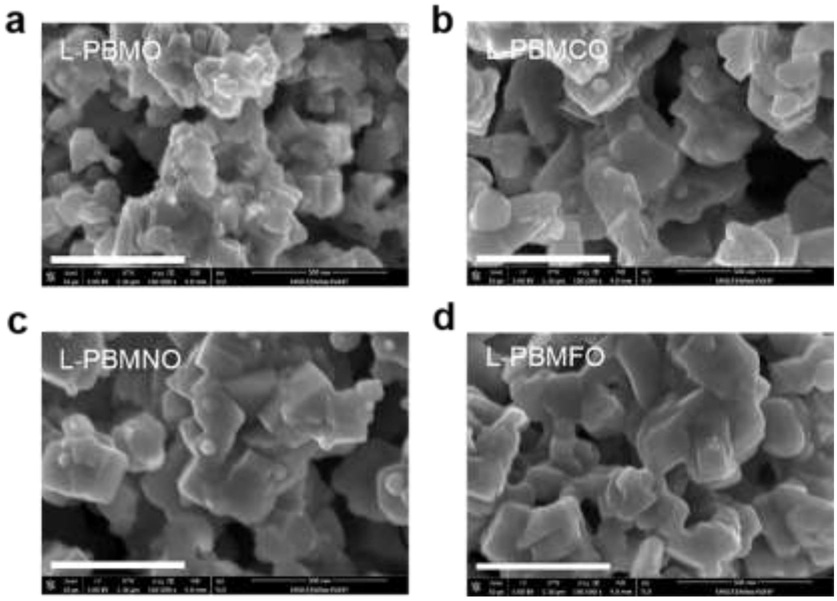

Supplementary Figure 3. Scanning electron microscope images of (a) L-PBMO, (b) L-PBMCO, (c) L-PBMNO, and (d) L-PBMFO after reducing treatments using a humidified (3% H_2O) H_2 at 800 ^oC; scale bar 500 nm.

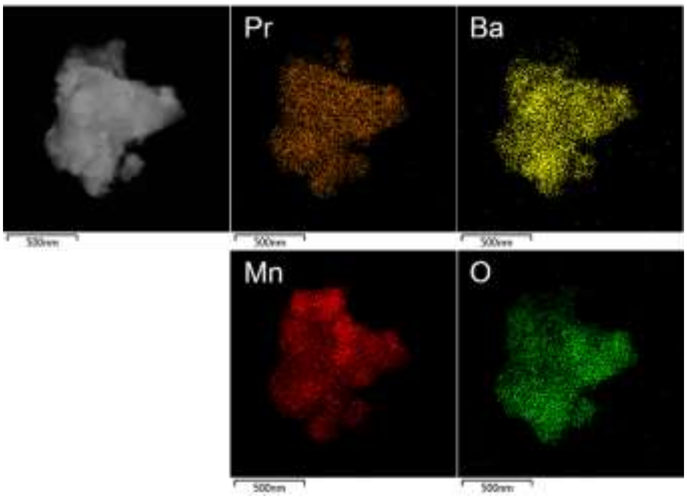

Supplementary Figure 4. X-ray diffraction patterns of (a) L-PBMO, (b) L-PBMCO, (c) L-PBMNO, and (d) L-PBMFO after reducing at 800 ^{o}C for 4h in humidified (3% H_2O) H_2

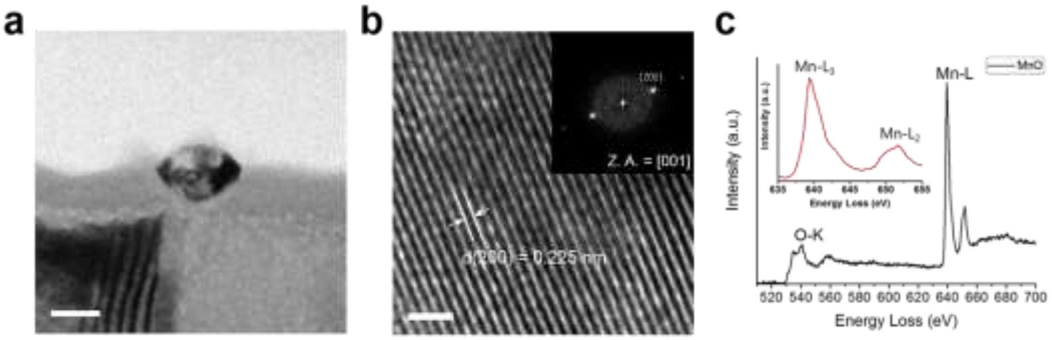

Supplementary Figure 5. (a) A bright-field (BF) TEM image of L-PBMO sample; scale bar 20 nm. (b) Magnified high-resolution TEM image; scale bar 1 nm and (c) Electron Energy-Loss Spectroscopy (EELS) analysis of exsolved MnO nanoparticle.

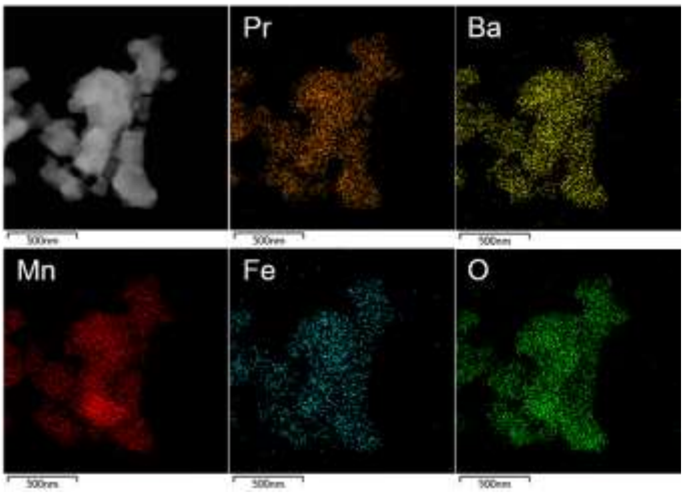

Supplementary Figure 6. Energy dispersive spectroscopy (EDS) micrographs and elemental mapping of Pr, Ba, Mn, Fe, and O, respectively, for L-PBMFO after reducing at 800 ^oC in humidified (3% H_2O) H_2.

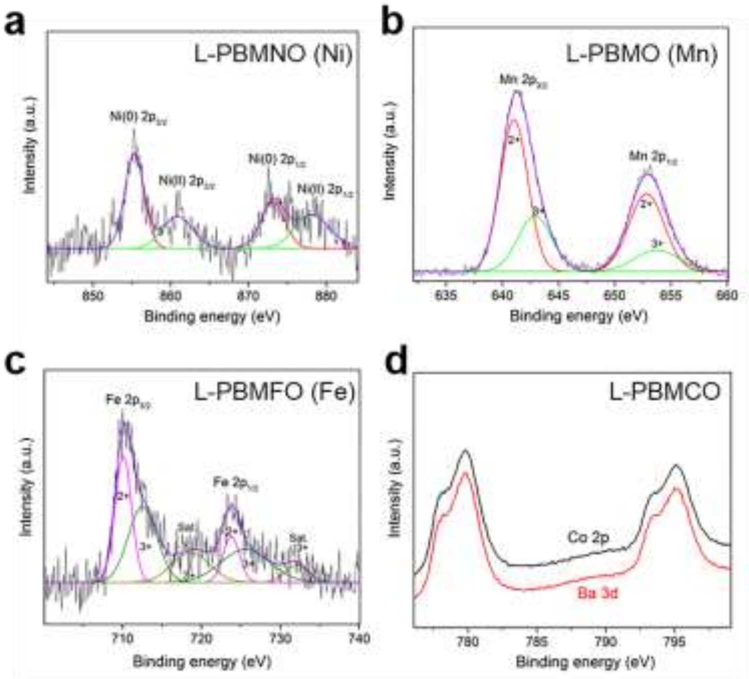

Supplementary Figure 7. XPS spectra of (a) Ni 2p in L-PBMNO, (b) Mn 2p in L-PBMO, (c) Fe 2p in L-PBMFO, and (d) Co 2p and Ba 3d in L-PBMCO.

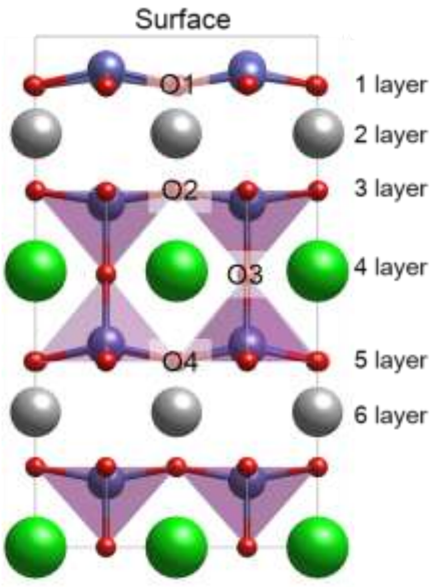

Supplementary Figure 8. Side view of L-PBMO. Possible vacancy positions except in the bottom two layers were considered to calculate vacancy formation energies (Table S1). The most stable vacancy sites at each layer's lattice oxygen are marked in each figure (O1-O4).

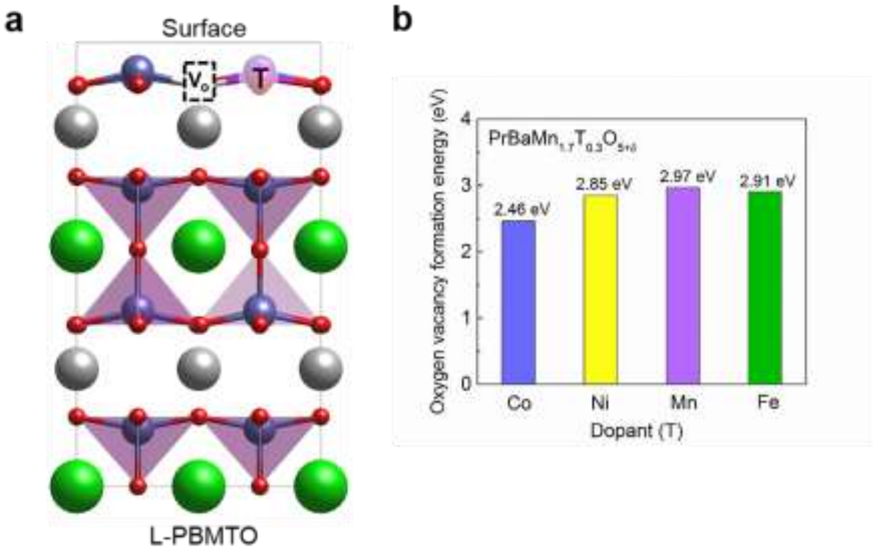

Supplementary Figure 9. Schematic of our model used for the calculations of oxygen vacancy formation on the surfaces. Pr, Ba, Mn, T (Mn, Co, Ni, and Fe), and O atoms are shown as gray, green, dark blue, purple, and red, respectively. The inset box indicates the oxygen vacancy.

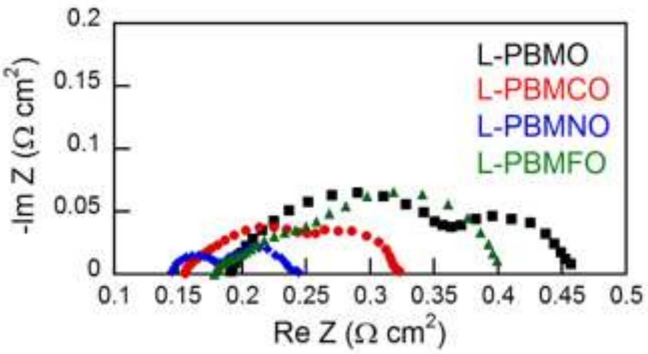

Supplementary Figure 10. Impedance spectra of the L-PBMTO (T = Mn, Co, Ni, and Fe) measured in humidified (3% H_2O) H_2 at 800 ^oC.

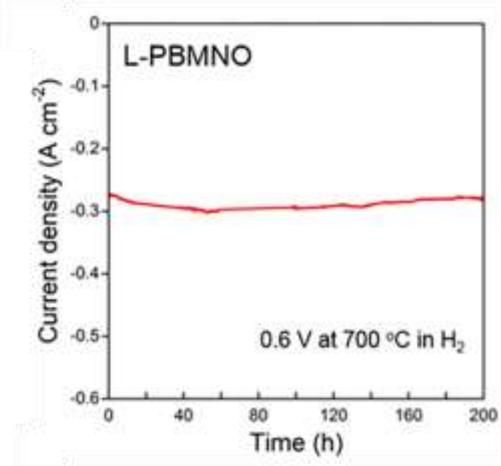

Supplementary Figure 11. Electrochemical performances of L-PBMNO anode in H_2 at 700 ^oC under a constant voltage of 0.6 V.

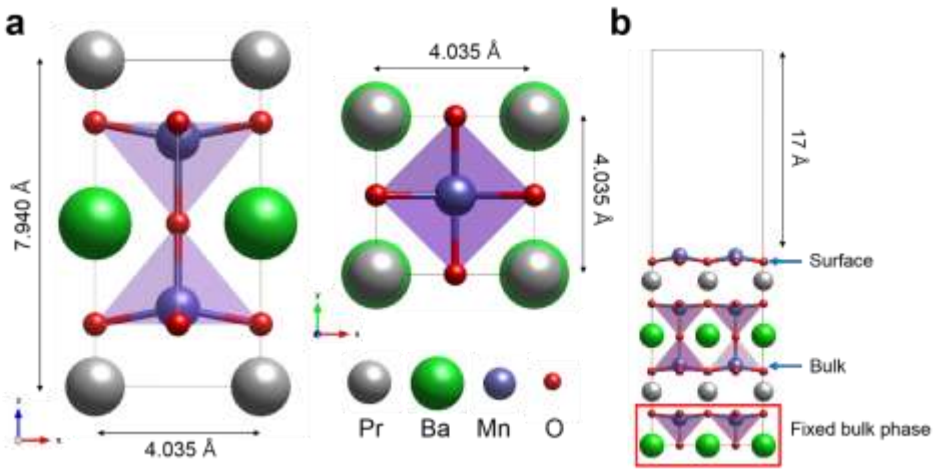

Supplementary Figure 12. Optimized (a) bulk and (b) surface structures of L-PBMO that we used for DFT calculations in this study.

Supplementary Table 1. Oxygen vacancy formation energies of L-PBMO at each layer.

	**Oxygen vacancy formation energy (eV)**
**1 layer**	2.97
**3 layer**	3.08
**4 layer**	3.72
**5 layer**	3.45

Supplementary Table 2. Comparison of the performance of SOFC in hydrocarbon reported in the present work and in the literature.

Cell configuration	Electrolyte thickness (μm)	Maximum power density (Wcm^{-2})	Temperature (°C)	Fuel	Ref
SFMNi/LDC/LSGM/LSCF	300	0.50	850	CH_4	1
A-LSCFe/YSZ/LSM	300	0.30	800	CH_4	2
LSCF/Ni–YSZ/YSZ/LSM	33	0.66	850	CH_4	3
PBMCo/YSZ/LSM	--	0.65	900	C_2H_4	4
K-PSCFN/LDC/LSGM/BCFN	300	0.60	850	CH_4	5
L-PBMCO/LDC/LSGM/NBSCF-GDC	250	0.33	800	C_3H_8	This Work
L-PBMNO/LDC/LSGM/NBSCF-GDC	250	0.32	800	C_3H_8	This Work

Supplementary Table 3. Comparison of the performance of SOFC at 800 ^{o}C in H_2 reported in the present work and in the literature.

Cell configuration	Electrolyte thickness (μm)	Maximum power density (Wcm^{-2})	Ref
SCMO/SDC/LSGM/SCF	300	0.660	6
LSCN/YSZ/YSZ-LSM	100	0.309	7
Mo-PBMO/GDC/YSZ/LSCF	100	0.560	8
SFMNi/LDC/LSGM/LSCF	300	0.792	1
SFNM/LSGM/PBCO	200	0.520	9
PSCFN/LDC/LSGM/BCFN	300	0.930	10
SMMO/SDC/LSGM/BSCF	280	0.659	11
L-PBMCO/LDC/LSGM/NBSCF-GDC	250	1.15	This work
L-PBMNO/LDC/LSGM/NBSCF-GDC	250	1.12	This work

Supplementary Table 4. Chemical compositions and abbreviations of samples.

Chemical composition	Abbreviation	Chemical composition	Abbreviation
$Pr_{0.5}Ba_{0.5}MnO_{3-\delta}$	S-PBMO	$PrBaMn_2O_{5+\delta}$	L-PBMO
$Pr_{0.5}Ba_{0.5}Mn_{0.85}Co_{0.15}O_{3-\delta}$	S-PBMCO	$PrBaMn_{1.7}Co_{0.3}O_{5+\delta}$	L-PBMCO
$Pr_{0.5}Ba_{0.5}Mn_{0.85}Ni_{0.15}O_{3-\delta}$	S-PBMNO	$PrBaMn_{1.7}Ni_{0.3}O_{5+\delta}$	L-PBMNO
$Pr_{0.5}Ba_{0.5}Mn_{0.85}Fe_{0.15}O_{3-\delta}$	S-PBMFO	$PrBaMn_{1.7}Fe_{0.3}O_{5+\delta}$	L-PBMFO

References

1. Du, Z. *et al.* High-Performance Anode Material $Sr_2FeMo_{0.65}Ni_{0.35}O_{6-\delta}$ with *In Situ* Exsolved Nanoparticle Catalyst. *ACS Nano.* **10**, 8660-8669 (2016).

2. Sun, Y-F., Li, J-H., Wang, M-N., Hua, B., Li, J., & Luo, J-L. A-site deficient chromite perovskite with in situ exsolution of nano-Fe: a promising bi-functional catalyst bridging the growth of CNTs and SOFCs. *J. Mater. Chem. A.* **3**, 14625-14630 (2015).

3. Chang, H., Chen, H., Shao, Z., Shi, J., Bai, J., & Li, S-D. In situ fabrication of $(Sr,La)FeO_4$ with CoFe alloy nanoparticles as an independent catalyst layer for direct methane-based solid oxide fuel cells with a nickel cermet anode. *J. Mater. Chem. A.* **4**, 13997-14007 (2016).

4. Sun, Y.-F. *et al.* New opportunity for in situ exsolution of metallic nanoparticles on perovskite parent. *Nano Lett*. **16**, 5303-5309 (2016).

5. Yang, C., Yang, Z., Jin, C., Xiao, G., Chen, F., Han, M. Sulfur-tolerant redox-reversible anode material for direct hydrocarbon solid oxide fuel cells. *Adv. Mater*. **24**, 1439-1443, (2012).

6. Wei, T., Zhang, Q., Huang, Y.-H. & Goodenough, J. B. Cobalt-based double-perovskite symmetrical electrodes with low thermal expansion for solid oxide fuel cells. *J. Mater. Chem.* **22**, 225-231 (2012).

7. Sun, Y. *et al.* A-site deficient perovskite: the parent for in situ exsolution of high-active, regenerable nano-particles as SOFCs anode. *J. Mater. Chem. A.* **3**, 11048-11056 (2015).

8. Sun, Y. F. *et al.* Molybdenum doped $Pr_{0.5}Ba_{0.5}MnO_{3-\delta}$ (Mo-PBMO) double perovskite as a potential solid oxide fuel cell anode material. *J. Power Sources* **301**, 237–241 (2016).

9. Ding, H., Tao, Z., Liu, S. & Yang, Y. A redox-stable direct-methane solid oxide fuel cell (SOFC) with $Sr_2FeNb_{0.2}Mo_{0.8}O_{6-\delta}$ double perovskite as anode material. *J. Power Sources* **327**, 573–579 (2016).

10. Yang, C. *et al.* *In situ* fabrication of CoFe alloy nanoparticles structured $(Pr_{0.4}Sr_{0.6})_3(Fe_{0.85}Nb_{0.15})_2O_7$ ceramic anode for direct hydrocarbon solid oxide fuel cells. *Nano Energy*. **11**, 704–710 (2015).

11. Jiang, L., Liang, G., Han, J. & Huang, Y. Effects of Sr-site deficiency on structure and electrochemical performance in Sr_2MgMoO_6 for solid-oxide fuel cell. *J. Power Sources* **270**, 441–448 (2014).
